# Association between demographic factors and prognosis in urothelial carcinoma of the upper urinary tract: a systematic review and meta-analysis

**DOI:** 10.18632/oncotarget.10708

**Published:** 2016-07-19

**Authors:** Hyung Suk Kim, Chang Wook Jeong, Cheol Kwak, Hyeon Hoe Kim, Ja Hyeon Ku

**Affiliations:** ^1^ Department of Urology, Dongguk University Ilsan Medical Center, Goyang, Korea; ^2^ Department of Urology, Seoul National University Hospital, Seoul, Korea

**Keywords:** urothelial carcinoma, urinary urinary tract, demographic, prognosis, survival

## Abstract

We aimed to assess the prognostic significance of demographic factors, including age, sex, performance status, smoking status, obesity, and race in upper urinary tract urothelial carcinoma (UTUC) patients treated with radical nephroureterectomy through a systematic review and meta-analysis. We conducted PubMed search for all articles published until December 2014 according to Preferred Reporting Items for Systematic Review and Meta-analysis (PRISMA) guidelines. Survival outcomes of interest were intravesical recurrence (IVR) free survival, progression free survival (PFS), cancer-specific survival (CSS), and overall survival (OS). Seventy-nine studies, including numbers of subjects ranging from 24 to 9899, met the inclusion criteria. Advanced age was significantly associated with worse PFS [hazard ratio (HR) 1.01] and OS (HR 1.05). The significant predictors of CSS were age (HR 1.02) and performance status (HR 1.35). Female gender (HR 0.81) and smoking (HR 1.38) were the significant predictors only for IVR free survival. No significant associations with survival outcomes were observed in obesity and race. Our study reveals that age is one of the most important demographic predictor of survival in UTUC. Also, male gender, poor performance status, and smoking are also significantly related to worse survival outcomes. However, large well-designed prospective studies are required to investigate the precise prognostic significance of demographics.

## INTRODUCTION

Upper urinary tract urothelial carcinoma (UTUC) is a relatively rare malignancy, accounting for only 5–10% of urothelial carcinomas (UCs) [1]. Radical nephroureterectomy (RNU) with ipsilateral bladder cuff excision remains the standard treatment for patients with large, multifocal or high-grade tumors [1]. Despite definitive surgery, UTUC has high potential for local and distant recurrence, especially in patients with advanced disease [2, 3].

The identification of new prognostic factors could help guide decisions regarding administration of adjuvant chemotherapy and selective enrolment into clinical trials of novel therapies. Tumor stage and grade have been documented as the major prognostic factors in patients with UTUC [1, 2]. In addition, tumor size, architecture, location and multiplicity as well as lymphovascular invasion (LVI) have been suggested as potential prognostic factors [1]. However, to date, the definite risk factors for recurrence and survival in patients who received RNU are still unclear.

Previous reports showed that demographic factors, such as age [3], gender [4], performance status [5], smoking status [6] obesity [7] and race [8] may have prognostic value in UTUC.

However, because of the rarity of the disease and the contradictory conclusions among previous studies, the prognostic significance of demographics has not been clearly established in UTUC. Our purpose was to assess the value of demographic data as prognostic factors for UTUC after RNU through a systematic review of the literature and meta-analysis of the available data.

## RESULTS

### Study population

Table 1 shows individual data on the characteristics of the included studies and patient populations. We included 79 studies, comprising patients from Asia, North America, and Europe, with numbers of patients ranging from 24 to 9899. The studies were published between 1994 and 2014 and the patient recruitment periods ranged from 1986 to 2013. The median age ranged from 61 to 73 years and the median follow-up time was between 17.8 and 84 months. Only one was a prospective study [9]. Other pathologic characteristics of the eligible studies are reported in Supplementary Table 1.

**Table 1:** Study and patient characteristics of the eligible studies

Study	Year	Country	Recruitment period	No. of patients	Median age, range (years)	No. of sex (male/female)	Median follow-up, range (months)	No. of NACH	No. of ACH	No. of ART
Park [11]	2004	Korea	1991–2001	86	59.5 (mean)	81/5	43.8, 5-140	0	14	5
Chen [12]	2005	Taiwan	1993–2003	111	70.5 (mean), 38-91	69/42	49.3 (mean), 1-136	NA	9	4
Ataus [13]	2006	Turkey	1993–2003	24	61, 34-74	19/5	34.8 (mean), 5-97	NA	NA	NA
Chung [14]	2007	Taiwan	1996–2006	150	NA	66/84	47.5, 3-121	NA	0	NA
Koda [15]	2007	Japan	1995–2005	106	70.4 (mean)	NA	17.5, 1-97	NA	25	NA
Akao [16]	2008	Japan	1992–2005	90	71	57/33	42, 2-179	NA	24	16
Berger [17]	2008	USA	1997–2005	100	73, 53-97	65/35	84, 24-120	NA	NA	NA
Li [18]	2008	Taiwan	1990–2005	260	65,23-87	125/135	56, 12-181	0	0	NA
Soga [19]	2008	Japan	1986–2005	46	NA	34/12	NA	NA	24	NA
Capitanio [20]	2009	multination	1987–2007	1249	27-97	846/403	NA	0	0	NA
Chung [21]	2009	Taiwan	1996–2006	76	66 (mean), 41-93	38/38	48, 15-88	NA	0	NA
Kamihira [22]	2009	Japan	1995–2005	1003	68.6 (mean), 27-92	718/285	20, 1-122	NA	181	NA
Favaretto (I) [23]	2010	USA	1995–2008	274	NA	NA	NA	NA	NA	NA
Favaretto (II) [24]	2010	USA	1995–2008	253	72, 64-77 (IQR)	159/94	48	0	NA	NA
Ishikawa [25]	2010	Japan	1990–2005	208	70, 39-90	139/69	44, 1-212	0	0	0
Kim [26]	2010	Korea	1986–2006	238	64.1 (mean), 25-91	164/74	53.4, 3-240	NA	NA	NA
Kobayashi [27]	2010	Japan	2000–2004	221	72, 46-92	153/68	38.4, 0.8-92.2	0	35	NA
Pieras [28]	2010	Spain	1990–2006	79	67, 65-69 (95% CI)	62/17	71, 59-84 (95% CI)	NA	NA	NA
Takaoka [29]	2010	Japan	1989–2007	60	64.7 (mean), 40-83	40/20	51.3, 1.5-223.8	0	28	NA
Cho [30]	2011	Korea	1994–2009	87	62.2, 33-85	64/23	32, 1-131	NA	19	5
Hou [31]	2011	Taiwan	2003–2007	192	69.8 (mean), 43-89	79/113	43.8 (mean), 3.8-84.8	NA	NA	NA
Ku [32]	2011	Korea	1991–2006	181	63, 36-90	142/39	37.5, 1-174	NA	48	NA
Walton [33]	2011	multination	1987–2008	773	68, 61-75 (IQR)	533/240	34, 15-65 (IQR)	0	66	NA
Ariane [34]	2012	France	1995–2010	609	69.8, 61.9-76	415/194	27, 10-48	0	NA	NA
Chen [35]	2012	China	2007–2009	85	NA	58/24	28, 5-60	NA	NA	NA
Chromecki [36]	2012	multination	1987–2007	2492	69.2, 54.1-84.2 (IQR)	1681/811	45, 0-101 (IQR)	NA	247	NA
Godfrey [37]	2012	USA	1990–2010	211	70 (mean)	124/87	27, 11-65.5 (IQR)	NA	NA	NA
Hirano [38]	2012	Japan	1995–2010	151	NA	121/30	24, 3-162	NA	51	NA
Kobayashi [39]	2012	Japan	2005–2009	288	71.4, 32-89	197/91	20.2, 3-61.6	0	47	0
Kuroda [40]	2012	Japan	1999–2010	121	68, 38-86	92/29	44.4 (mean), 3.5-144.5	0	29	NA
Liang [41]	2012	Taiwan	1996–2004	340	68, 34-87	182/158	38, 1-176	0	20	4
Cho [42]	2013	Korea	1994–2009	78	61.1 (mean), 33-85	58/20	34, 12-132	0	NA	NA
Ehdaie [43]	2013	USA	1995–2008	288	71, 37-90	187/101	4.02 yr, 0.03-14.65 yr	NA	NA	NA
Elalouf [44]	2013	France	1998–2011	237	69.3, 60-76 (IQR)	161/76	44, 24-79 (IQR)	0	23	NA
Fairey [45]	2013	Canada	1994–2009	849	NA	542/307	2.2 yr, 0.6-5 yr	NA	94	NA
Fujita [46]	2013	Japan	1999–2011	139	72, 48-90	91/48	27, 1-139	11	23	NA
Gunay [47]	2013	Turkey	1987–2009	101	60.5 (mean)	85/16	56.2 (mean)	NA	NA	NA
Hashimoto [48]	2013	Japan	1997–2010	84	68.8	59/25	NA	NA	NA	NA
Ito [49]	2013	Japan	2005–2008	72	NA	43/29	24.9, 2.6-39.3	0	14	NA
Kim [50]	2013	Korea	2000–2013	65	60.4 (mean), 37-87	40/25	34, 12-114	NA	36	NA
Kim [51]	2013	Korea	1990–2010	422	64, 29-86	318/104	44, 3-257	NA	51	NA
Kusuda [52]	2013	Japan	2000–2009	502	NA	360/142	39 (mean), 6-134.8	NA	NA	NA
Milojevic [53]	2013	Serbia	1999–2010	183	66 (mean), 36-88	102/81	35, 1-154	0	NA	NA
Morizane [54]	2013	Japan	1995–2011	99	73, 44-86	71/28	37.9, 6.6-171.4	NA	33	NA
Rink [55]	2013	multination	1987–2007	864	70, 61-76 (IQR)	553/311	50, 23-90 (IQR)	0	63	0
Sakano [56]	2013	Japan	1995–2009	536	71, 32-93	370/166	40.9, 3-200	32	NA	NA
Shimamoto [57]	2013	Japan	1983–2008	105	66.9, 42-88	74/31	53 (mean)	7	59	NA
Takahara [58]	2013	Japan	1996–2009	103	68.6 (mean), 62-75 (IQR)	71/32	29, 14-63 (IQR)	NA	12	NA
Xylinas [59]	2013	France	1995–2010	482	69.2, 60-76 (IQR)	332/150	39.5, 25-60 (IQR)	0	59	NA
Zhang [60]	2013	China	2000–2010	217	69, 62-81	130/87	52, 12-78 (IQR)	0	NA	NA
Aziz [61]	2014	Germany	1990–2012	265	NA	169/96	23, 9-48 (IQR)	0	47	NA
Bachir [62]	2014	Canada	1990–2010	644	NA	NA	24.5	28	76	NA
Cho [63]	2014	Korea	2004–2012	147	70, 44-84	41/106	33, 1-191	0	95	NA
Choo [64]	2014	Korea	2000–2011	319	NA	253/66	NA	6	85	NA
Ehdaie [65]	2014	USA	1995–2008	253	72, 63-77 (IQR)	158/95	NA	0	0	NA
Fang [66]	2014	China	2000–2010	438	66, 20-88	187/251	45, 12-144	0	NA	NA
Fradet [67]	2014	Canada	1990–2010	743	69.7 (mean)	438/304	24.8, 7.7-56.8 (IQR)	0	73	NA
Fujita [68]	2014	Japan	1998–2012	226	70, 37-90	153/73	41, 1-164	8	44	NA
Gandaglia [69]	2014	USA	1988–2009	9899	73, 22-99	5823/4076	98, 0-263	NA	NA	NA
Ichimura [70]	2014	Japan	1996–2012	171	NA	119/52	56, 25-86 (IQR)	0	NA	NA
Ishioka (I) [71]	2014	Japan	1995–2010	1014	70, 62-79 (IQR)	718/296	38, 16-73 (IQR)	NA	155	NA
Ishioka (II) [72]	2014	Japan	1995–2010	754	69, 62-75 (IQR)	526/228	41, 18-75 (IQR)	NA	NA	NA
Ito [73]	2014	Japan	1999–2012	70	NA	47/23	29.2, 1-157	NA	9	NA
Kitamura [74]	2014	Japan	1995–2010	110	NA	NA	60, 6-192	NA	NA	NA
Kondo [75]	2014	Japan	1988–2013	180	NA	128/52	NA	NA	NA	NA
Lee [76]	2014	Taiwan	2004–2010	250	NA	108/142	41	NA	NA	NA
Liu (I) [77]	2014	China	1998–2009	230	NA	179/51	67, 34-170	0	57	NA
Liu (II) [78]	2014	China	2002–2010	212	66.5, 31-84	123/89	39, 7-78	0	8	NA
Ou [79]	2014	Taiwan	2003–2011	61	NA	34/27	39.7, 12-96	0	0	0
Park [80]	2014	Korea	1991–2010	392	64, 29-86	299/93	47.6, 2-257	0	60	NA
Sasaki [81]	2014	Japan	1996–2012	171	NA	119/52	NA	0	NA	NA
Shirotake [82]	2014	Japan	1993–2011	873	70, 63-77 (IQR)	608/231	32, 16-62 (IQR)	0	129	NA
Sung (I) [83]	2014	Korea	1994–2011	410	64, 55-72 (IQR)	312/98	40.2, 33-66.1 (IQR)	NA	91	NA
Sung (II) [84]	2014	Korea	1994–2010	386	NA	293/93	39, 21.1-70.6 (IQR)	NA	NA	NA
Tanaka [85]	2014	Japan	1994–2010	474	69, 61-76 (IQR)	346/128	35, 17-68 (IQR)	0	78	NA
Xylinas (I) [86]	2014	multination	1987–2007	519	70, 61-76 (IQR)	330/189	37, 19-73 (IQR)	0	53	0
Xylinas (II) [87]	2014	multination	1987–2007	2681	68.4, 54-84	1808/873	57.5, 1-271	0	264	NA
Yafi [88]	2014	Canada	1995–2010	1029	70, 62-77 (IQR)	195/113	17.8, 5.5-46.8	NA	36	NA
Zou [89]	2014	China	1999–2013	122	64, 35-80	87/35	53, 3-159	NA	NA	NA

NACH: neoadjuvant chemotherapy, ACH: adjuvant chemotherapy, ART: adjuvant radiotherapy, NA: not available, IQR: interquartile range, CI: confidence interval.

### Demographic predictors of outcomes

### Age

**Figure 1:**
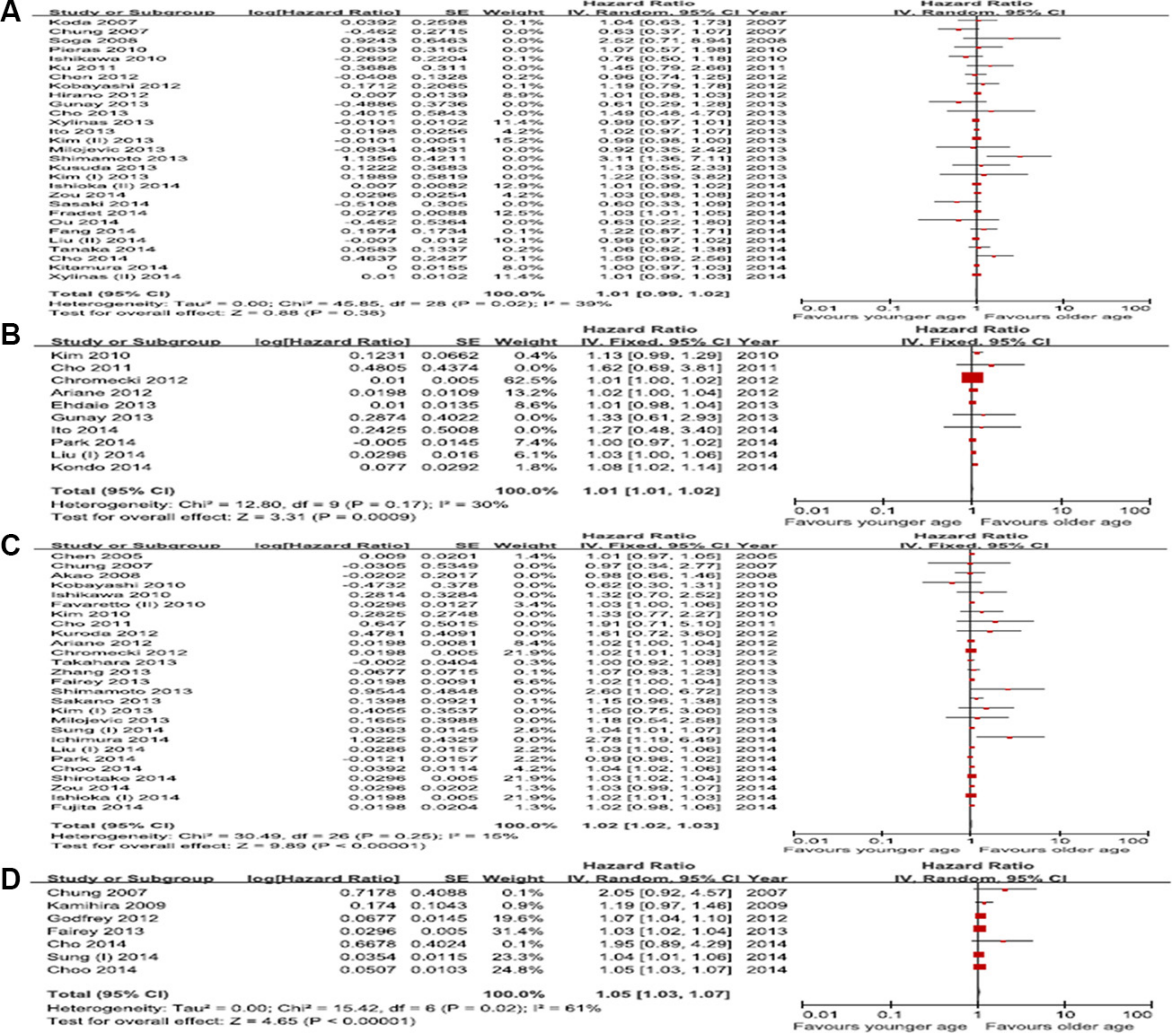
Forest plots of prognosis of age (**A**) Intravesical recurrence-free survival, (**B**) Progression-free survival, (**C**) Cancer-specific survival, (**D**) Overall survival.

In the 29 studies about intravesical recurrence (IVR)-free survival, there was an obvious inter-study heterogeneity (*p* = 0.02); thus, the random effect model was used to pool the results. The meta-analysis showed no association between age and IVR-free survival (hazard ratio (HR), 1.01; 95% confidence interval (CI), 0.99–1.02; *p* = 0.38) (Figure 1A). In the 10 studies about progression free survival (PFS) there was no obvious heterogeneity among studies (*p* = 0.17; *I*^2^ = 30%); thus, the fixed effects model was used to pool the results. The pooled HR for PFS was 1.01 (95% CI, 1.01–1.02; *p* = 0.0009) (Figure 1B), indicating a significant association between age and PFS. A total of 27 studies were included in the analysis of cancer-specific survival (CSS). Age was associated with worse CSS (HR, 1.02; 95% CI, 1.02–1.03; *p* < 0.00001). Since inter-study heterogeneity was not significant (*p* = 0.17; *I*^2^ = 30%), the fixed effects model was applied (Figure 1C). Seven studies were included in the analysis of overall survival (OS). Age was associated with worse OS (HR, 1.05; 95% CI, 1.03–1.07; *p* < 0.00001). There was significant heterogeneity (*p* = 0.02; *I*^2^ = 61%) (Figure 1D).

### Gender

**Figure 2:**
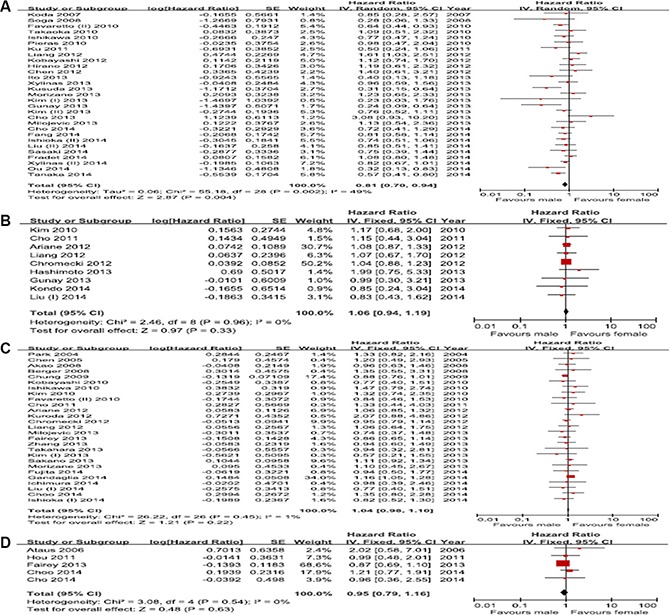
Forest plots of prognosis of sex (**A**) Intravesical recurrence-free survival, (**B**) Progression-free survival, (**C**) Cancer-specific survival, (**D**) Overall survival.

Twenty-nine studies on the relationship between sex and IVR were included. The present meta-analysis indicated that female patients had a better IVR-free survival rate than male patients (HR, 0.81; 95% CI, 0.70–0.94; *p* = 0.004). However, the Cochrane *Q* test (*p* = 0.002) could not exclude a significant heterogeneity (Figure 2A). Nine studies provided information on PFS. There was no significant association between sex with PFS (HR, 1.06; 95% CI, 0.94–1.19; *p* = 0.33), and no heterogeneity was detected in the data (*p* = 0.96; *I*^2^ = 0%) (Figure 2B). A total of 27 studies evaluated the relationship between sex and CSS. The pooled HR (95% CI) of these studies for CSS was 1.04 (0.98–1.10; *p* = 0.22), and there was no heterogeneity (*p* = 0.45; *I*^2^ = 1%) (Figure 2C). The meta-analysis of 5 studies also demonstrated no significant association between sex and overall survival (OS) (HR, 0.95; 95% CI, 0.79–1.16; *p* = 0.63) in a fixed effects model (*p* = 0.54, *I*^2^ = 0%) (Figure 2D).

### Performance status

**Figure 3:**
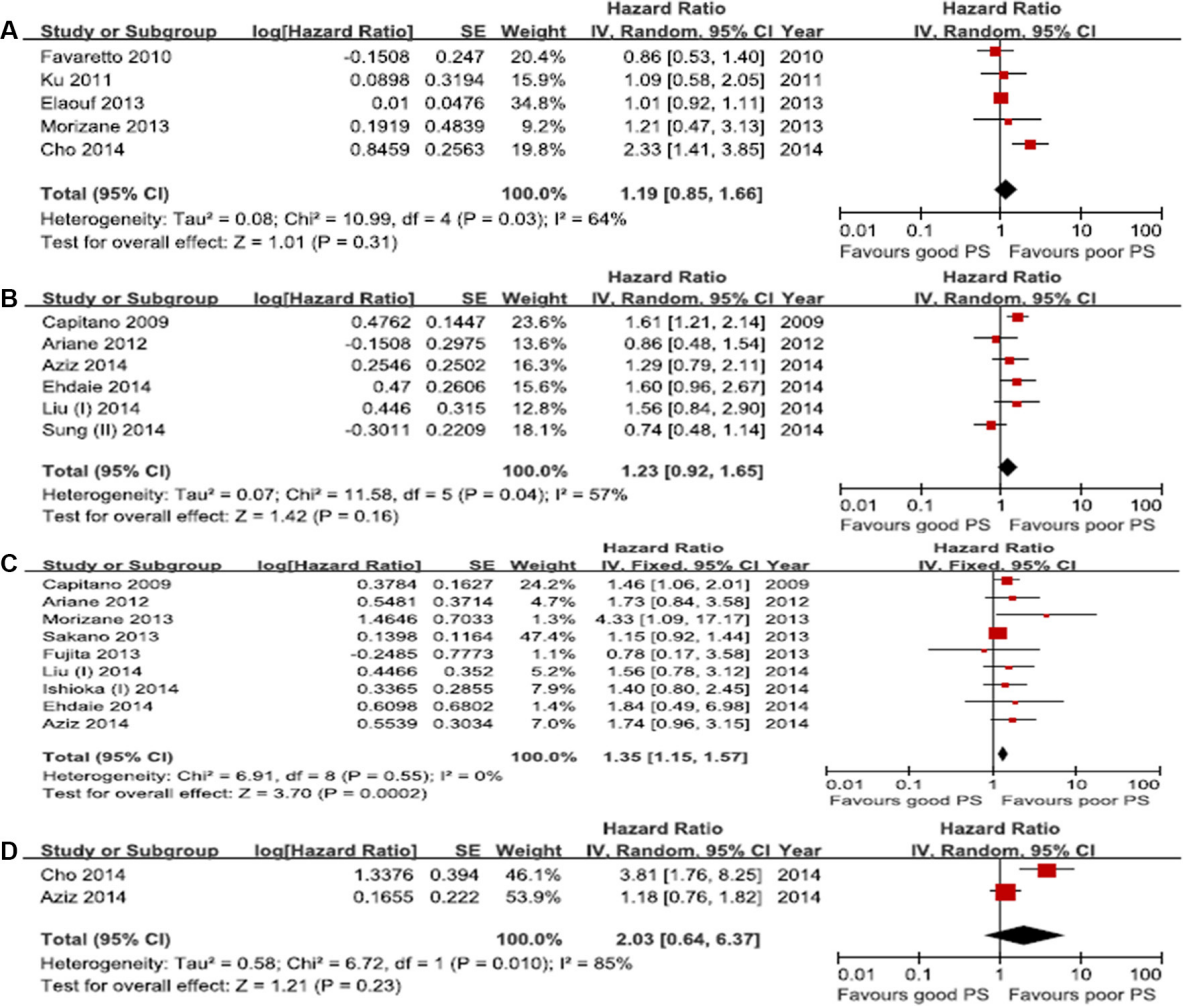
Forest plots of prognosis of performance status (**A**) Intravesical recurrence-free survival, (**B**) Progression-free survival, (**C**) Cancer-specific survival, (**D**) Overall survival.

Four studies were included in the analysis of the correlation between performance status and IVR-free survival. The results indicated that performance status had no relationship with IVR-free survival (HR, 1.19; 95% CI, 0.85–1.66; *p* = 0.31), and there was between-study heterogeneity (*p* = 0.03; *I*^2^ = 64%) (Figure 3A). The pooled analysis of PFS was based on 6 studies. No significant association between performance status and PFS was observed (pooled HR, 1.74; 95% CI, 0.92–1.65; *p* = 0.16). The test for heterogeneity recorded an *I*^2^ value of 57% (*p* = 0.04), suggesting the presence of inter-study heterogeneity (Figure 3B). Nine eligible studies were analyzed to evaluate the impact of performance status on CSS, and the analysis revealed that performance status was significantly related to CSS (HR, 1.35; 95% CI, 1.15–1.57; *p* = 0.0002). No significant heterogeneity was observed (*p* = 0.05; *I*^2^ = 0%) (Figure 3C). The pooled HR estimate for OS was 2.03 (95% CI, 0.64–6.37; *p* = 0.23) in the analysis of 2 studies, indicating no clear correlation between performance status and OS. The result for the test for heterogeneity was significant (*p* = 0.01; *I*^2^ = 85%) (Figure 3D).

### Smoking history

**Figure 4:**
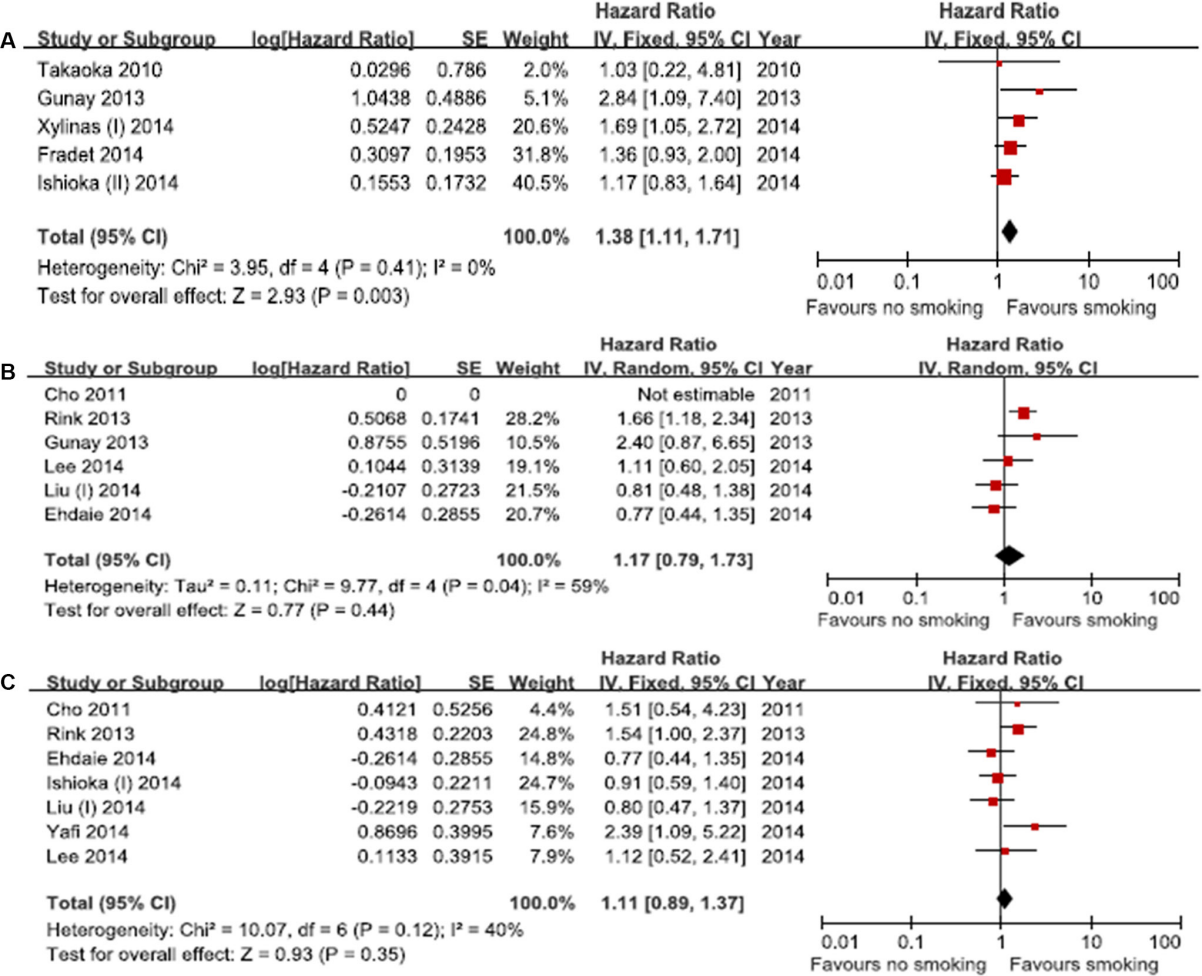
Forest plots of prognosis of smoking (**A**) Intravesical recurrence-free survival, (**B**) Progression-free survival, (**C**) Cancer-specific survival.

Data on IVR-free survival was available from 5 studies. Smoking history was associated with a higher risk of IVR (HR, 1.38; 95% CI, 1.11–1.71; *p* = 0.003). Importantly, this analysis revealed no heterogeneity (*p* = 0.41; *I*^2^ = 0%) (Figure 4A). Six studies provided data on PFS. The results suggested that smoking status did not correlate with poor PFS (HR, 1.17; 95% CI, 0.79–1.73; *p* = 0.44), but there was significant heterogeneity (*I*^2^ = 59%) (Figure 4B). CSS rate was extracted from 7 studies. The meta-analysis indicated no significant association between smoking history and poor CSS (HR, 1.1; 95% CI, 0.89–1.37; *p* = 0.35). No significant heterogeneity was observed in the primary analysis (*p* = 0.12; *I*^2^ = 40%) (Figure 4C). The meta-analysis was not performed for OS because only 1 study was eligible for inclusion [10].

### Obesity

In 3 studies about IVR-free survival, the pooled HR for IVR-free survival showed that obesity was not significantly associated with IVR-free survival (HR, 1.07; 95% CI, 0.65–1.75; *p* = 0.80). However, significant heterogeneity was observed (*I*^2^ = 64%) (Supplementary Figure 1A). Of 2 studies about PFS, the pooled HR for PFS was 0.98 (95% CI, 0.47–2.06; *p* = 0.96), which suggested no significant correlation between obesity and PFS. There was no significant heterogeneity among these studies (*p* = 0.46; *I*^2^ = 0%) (Supplementary Figure 1B). Four studies reported survival analysis for CSS. Obesity was not significantly related to worse CSS (HR, 0.99; 95% CI, 0.96–1.02; *p* = 0.38). However, there was an obvious inter-study heterogeneity (*I*^2^ = 53%) (Supplementary Figure 1C). Two studies were included in the analysis of OS. Obesity did not exhibit a correlation with worse OS (HR, 0.94; 95% CI, 0.80–1.19; *p* = 0.43). The inter-study heterogeneity was significant (*I*^2^ = 51%) (Supplementary Figure 1D).

### Race

No studies were available for investigating the association between race and IVR-free survival. Furthermore, we could not perform meta-analysis for PFS and OS because only 1 study was eligible for PFS [11] and OS [12], respectively. Only 2 studies were evaluated for the impact of race on CSS. The pooled HR value was 1.02 (95% CI, 0.88–1.18; *p* = 0.79), which indicated no significant association between race and CSS. There was no heterogeneity between studies (*p* = 0.21; *I*^2^ = 37%) (Supplementary Figure 2).

### Publication bias

Due to the small number of studies in most meta-analyses, the potential for publication bias could be reliably examined only in the meta-analyses of the correlation between age and IVR-free survival, PFS, and CSS, and between sex and IVR-free survival and CSS, which included ≥ 10 studies (Supplementary Figures 3 and 4). We found no strong evidence for publication bias by graphical inspection. However, Egger's test suggested there was a significant publication bias in the meta-analysis of the correlation between age and PFS (*p* = 0.03045). No publication bias was detected in the other meta-analyses (all *p* > 0.05, Egger's test).

## DISCUSSION

UTUC is a heterogeneous disease showing a variety of clinical courses. In spite of the increased detection of earlier-stage tumors resulting from the recent improvement in imaging and endoscopic techniques, UTUC still remains an aggressive disease with high recurrence and progression rates [1]. According to the current European Association of Urology (EAU) guidelines, the prognostic predictors of UTUC can be largely divided into preoperative clinical (demographic) and postoperative pathological factors [1, 2]. Many studies have focused mainly on pathological factors, including tumor stage, tumor grade, concomitant CIS, lymph node invasion, tumor multifocality, tumor architecture, tumor necrosis, and LVI. In contrast, the studies mainly evaluating demographic factors, such as age, sex, obesity, performance status, smoking, and race, are relatively insufficient and have shown conflicting results; therefore, the prognostic significance of demographic data has not been definitely confirmed in UTUC. However, knowledge of the prognostic significance of preoperative demographic factors is important for the counselling and management of UTUC patients.

We performed a systematic review and meta-analysis to assess whether each demographic factor could predict the survival outcomes in patients treated with RNU for UTUC. This study aggregated the outcomes of 79 studies with numbers of patients ranging from 24 to 9899. On the basis of the available data, a total of 20 meta-analyses were conducted to investigate the association of each demographic factor with survival outcomes. To the best of our knowledge, this is the first study to systematically evaluate the association between each demographic factor and postoperative survival outcomes, including IVR-free survival, PFS, CSS, and OS. Our meta-analysis found an association between survival outcomes of UTUC and demographic factors. Advanced age was significantly associated with worse PFS, CSS, and OS, but not with IVR-free survival. Male sex and smoking history only correlated with poor IVR-free survival. Performance status was exclusively related to CSS. Obesity and race did not show any association with survival outcomes.

There are several limitations for this systematic review and meta-analysis. First, because we did not use sample-size restrictions, most meta-analyses included a small number of studies (less than 10). As a result, the evaluation for publication bias was possible only in 5 meta-analyses including more than 10 studies (effect of age on IVR-free survival, PFS, and CSS, and of sex on IVR-free survival and CSS). However, among the meta-analyses assessed for publication bias, no significant bias was observed (all *p* > 0.05 for Egger's test), except for the meta-analysis of the effect of age on PFS. Second, owing to scarcity or absence of data reported in the literature, the association between smoking and race and several survival outcomes (smoking and OS, race and IVR free survival, PFS, and PS) could not be evaluated in this meta-analysis. Third, there was a significant heterogeneity among studies included in half of the current meta-analyses. The meta-regression and subgroup analysis to identify the source of inter-study heterogeneity was not carried out in the present study. Although the random-effects model, which accounts for heterogeneity, was applied to analyze the studies with heterogeneity, the conclusions yielded in this systematic review and meta-analysis should be interpreted with caution [13, 14]. Fourth, the results of this systematic review and meta-analysis were partially drawn on the basis of unadjusted estimates from Kaplan-Meier or univariate Cox regression analyses because some studies did not provide detailed information. Furthermore, it should be kept in mind that a possible bias [15] may be due to the retrospective nature of almost all studies analyzed, except for one [9]. To the best of our knowledge, high quality prospectively, randomized controlled trials investigating the association between demographic factors and prognosis in UTUC have not been published to date. Finally, we cannot exclude the selection bias by including only English language written articles.

This first meta-analysis has yielded significant associations between demographic factors and IVR, progression and mortality in patients with UTUC who underwent RNU, although these findings need to be interpreted with caution. Based on a meta-analysis of the available data, age is one of the most important demographic marker, and is independently associated with mortality. We also identified male sex and smoking history as significant predictors of IVR. However, since the current evidence lacks prospective evaluation of this relationship, large, well-designed prospective studies are required to investigate the precise prognostic significance of demographic factors.

## MATERIALS AND METHODS

A systematic review was performed in accordance with Cochrane Collaboration and Preferred Reporting Items for Systematic Review and Meta-analysis (PRISMA) guidelines [16].

### Search strategy

PubMed was searched for all articles published till December 2014 on the topic of interest. Search terms used were (“nephroureterectomy”) and (“cancer” OR “carcinoma”). Article selection was performed by two independent evaluators (H.S.K., C.W.J.), and all the discrepancies between the two were resolved.

### Inclusion and exclusion criteria

According to the PRISMA guidelines, we used the Population, Intervention, Comparator, Outcome, and Study design (PICOS) approach to define study eligibility [16].

Population: patients with UTUC.

Intervention: RNU.

Comparator: demographic data including age, sex, performance status, smoking status, obesity, and race.

Outcome: IVR-free survival, PFS, CSS, and OS.

Study design: univariate and/or multivariate Cox regression analysis.

The inclusion criteria were: (1) original article, (2) human research, (3) English language, (4) UTUC, (5) treatment exclusively with RNU, and (6) availability of Kaplan-Meier/Cox regression-derived results for the prognostic value of demographics on UTUC outcomes according to the REMARKS guidelines for assessment of prognostic markers [17]. No sample-size restrictions were used. Studies using analyses other than survival analysis were not included. In addition, studies which did not offer sufficient data to acquire the hazard ratio (HR) and its standard error (SE) were excluded. If duplicate study populations or analyses of repeated data were identified, only the most recent or most complete study was preferentially assessed.

In the present meta-analysis, IVR-free survival was defined as the interval between surgery and the occurrence of UC in the bladder. PFS was defined as the interval between surgery and the subsequent appearance of local failure, either at the operative site or in the regional lymph nodes, or distant metastasis, while recurrence in the bladder was not included in PFS analysis. We excluded from PFS analysis the studies in which PFS was defined as recurrence both in bladder and non-bladder lesions. CSS and OS were defined as the interval between surgery and death from UTUC or death from any cause, respectively.

### Data extraction and analysis

**Figure 5:**
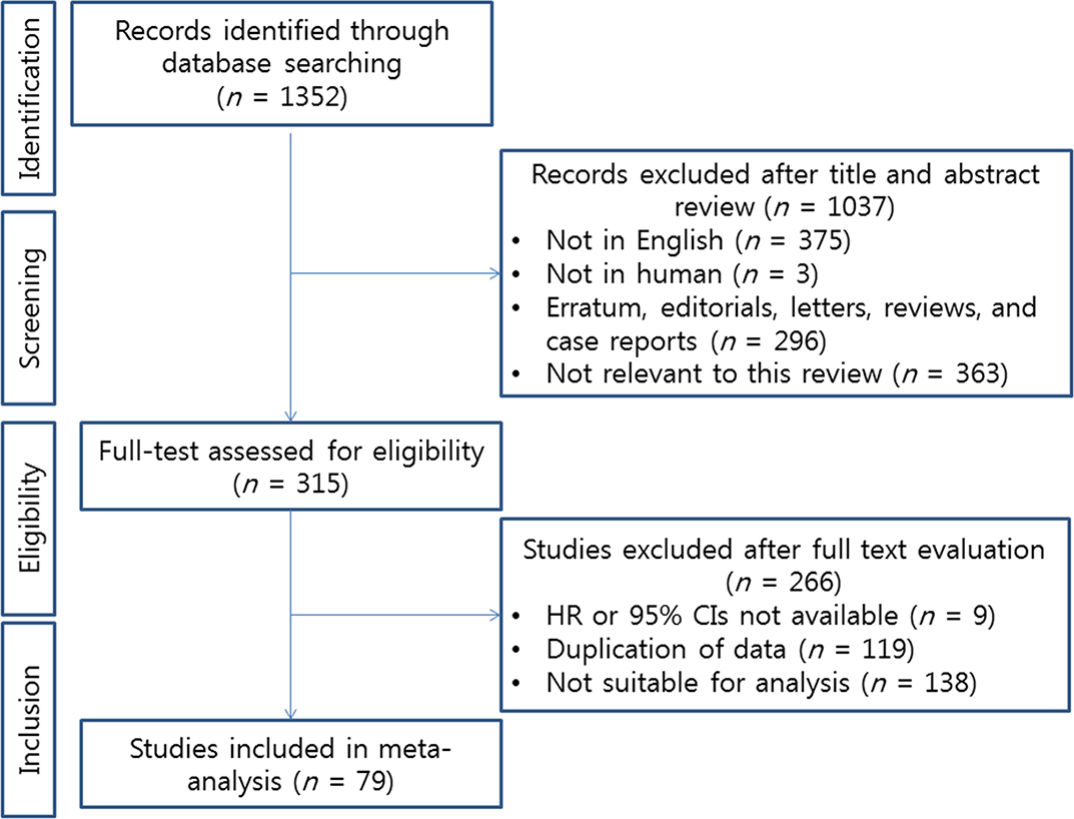
Preferred reporting items for systematic reviews and meta-analysis (PRISMA) flow chart

Two authors (C.K. and H.H.K.) performed an independent review of 1,352 articles. A total of 1,037 articles were excluded upon examination of the titles and abstracts, while the full text of 315 articles was evaluated. In accordance with all previously mentioned inclusion criteria, a final selection of 79 articles was made [9–12, 18–92]; any discrepancies between the two evaluations were resolved. The PRISMA flow chart depicting the process for the systematic literature search and selection of the studies is shown in Figure 5.

Data was independently extracted by two authors (H.S.K. and J.H.K.) from all the included studies and subsequently crosschecked to ensure accuracy. Any discrepancies between the two authors were resolved. Information was retrieved according to the REMARKS guidelines for reporting prognostic markers, including author, country, journal, publication year, prognostic factors examined, study design, study population (sample size, recruitment period, and follow-up), patient characteristics, treatment received, statistical method applied (with variables used for adjustment), and reported impact of examined factors on UTUC outcome using univariate or multivariate analyses.

### Meta- analysis

We conducted a meta-analysis to summarize quantitatively the overall prognostic value of demographic factors. The cumulative effects of factors of interest were evaluated by the inverse variance method. If the 95% CI was not reported, due to the paucity of prognostic literature directly reporting these values, previously reported indirect methods were utilized to extract the log HR and variance [93]. Either the fixed-effects or the random-effects model was used, in case of absence or presence of between-study heterogeneity, respectively. Statistical heterogeneity was assessed using both the Cochran *Q* test and the *I*^2^ statistic, which describes the percentage of total variation across studies caused by heterogeneity rather than by chance. A value of *p* < 0.05 for the Cochran *Q* test or an *I*^2^ statistic > 50% indicated the presence of significant heterogeneity across selected studies [13, 14] which resulted in the use of the random-effects model based on the Der Simonian method for estimating the tau value [13]. To assess the risk of publication bias, we used a funnel plot and the Egger test for outcomes when at least 10 statistically significant studies were included in the meta-analysis [15]. The meta-analysis was performed using RevMan 5.0 statistical software (the Cochrane Collaboration, Copenhagen).

## SUPPLEMENTARY MATERIALS FIGURES AND TABLES




