# Supplementary material for: Association between demographic factors and prognosis in urothelial carcinoma of the upper urinary tract: a systematic review and meta-analysis
Source: Oncotarget. 2016 Jul 19;8(5):7464–76. doi: 10.18632/oncotarget.10708 (PMC5352335; doi:10.18632/oncotarget.10708)
Supplement: Supplementary file 1 [file oncotarget-08-7464-s001.pdf]

Association between demographic factors and prognosis in urothelial carcinoma of the upper urinary tract: a systematic review and meta-analysis

Supplementary Materials

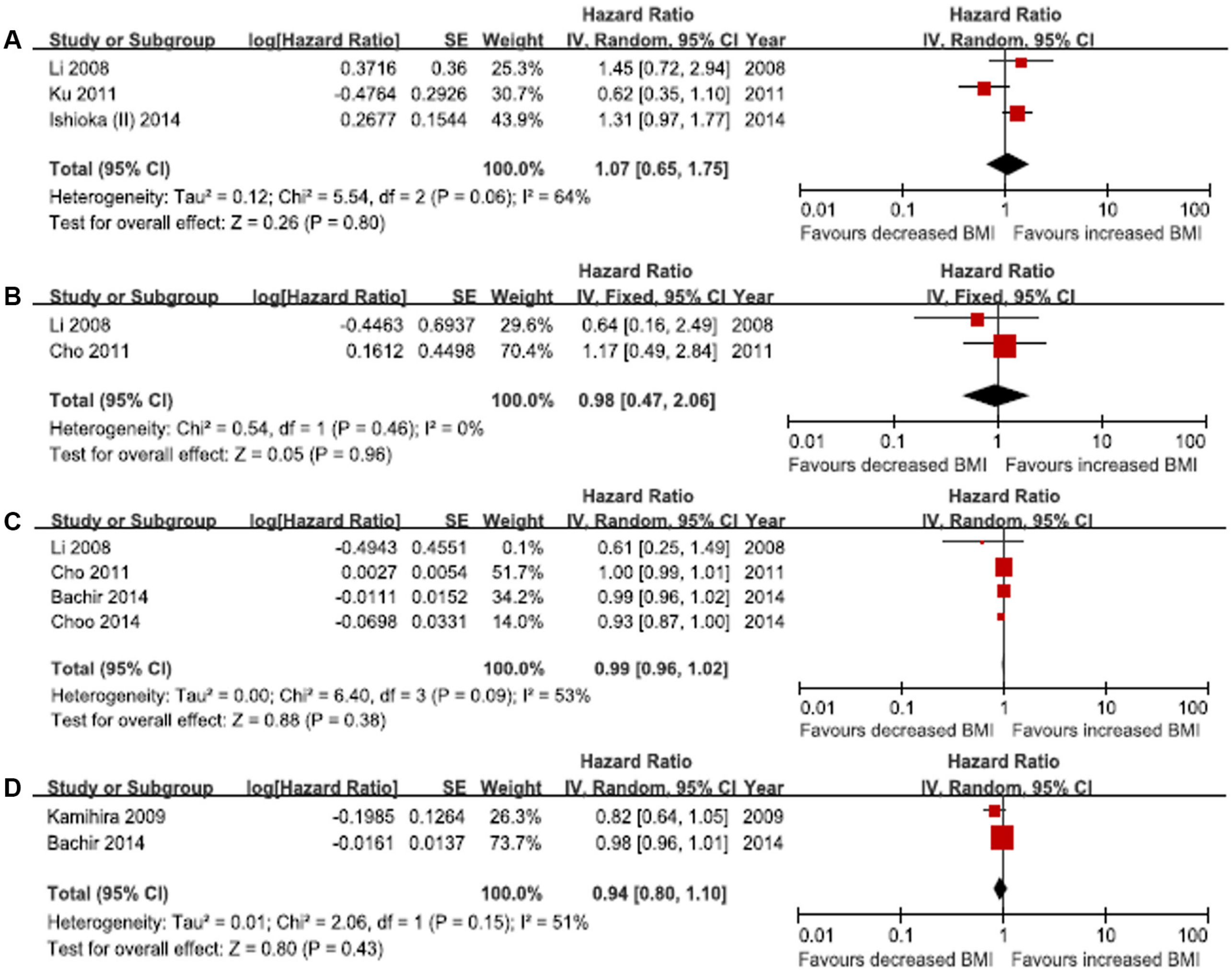

Supplementary Figure S1: Forest plots of prognosis of obesity. (**A**) Intravesical recurrence-free survival, (**B**) Progression-free survival, (**C**) Cancer-specific survival, (**D**) Overall survival.

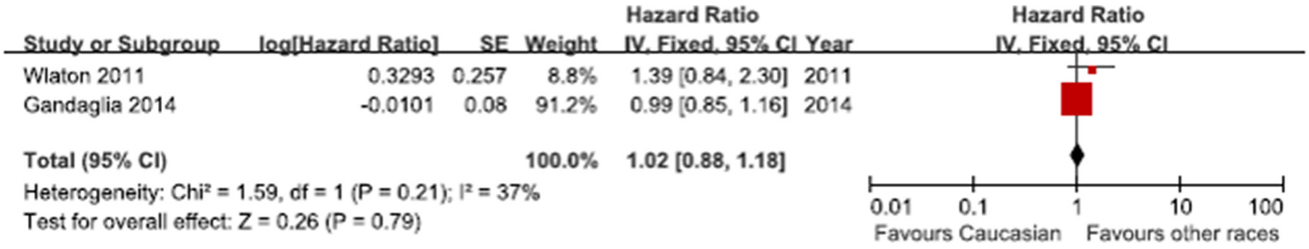

Supplementary Figure S2: Forest plots of cancer-specific survival of race.

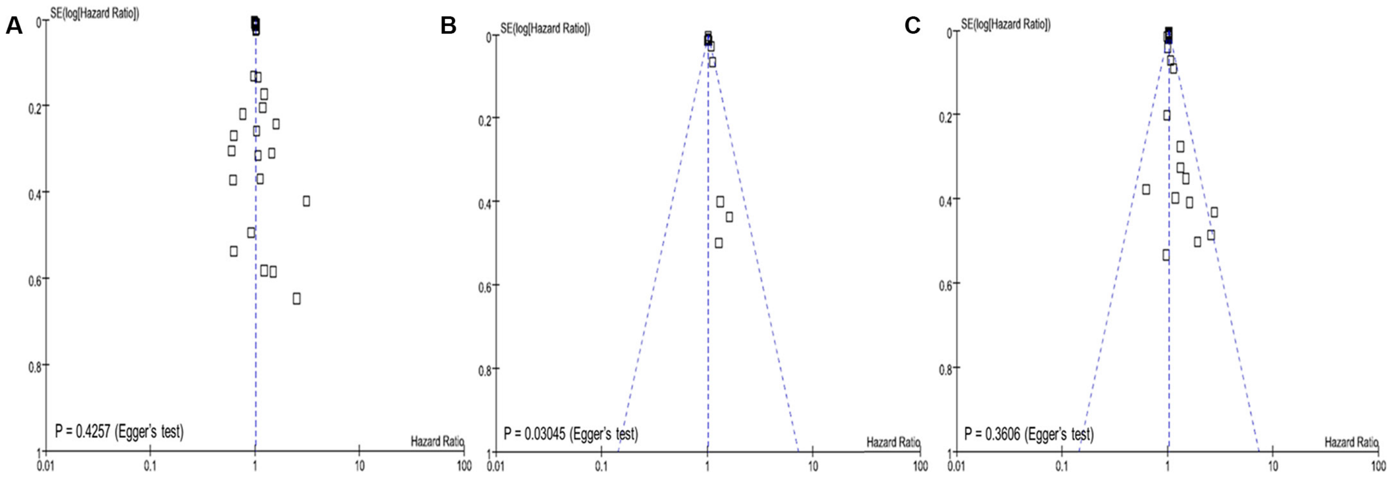

Supplementary Figure S3: Funnel graphs of the assessment of potential publication bias of age. (**A**) Intravesical recurrence-free survival, (**B**) Progression-free survival, (**C**) Cancer-specific survival.

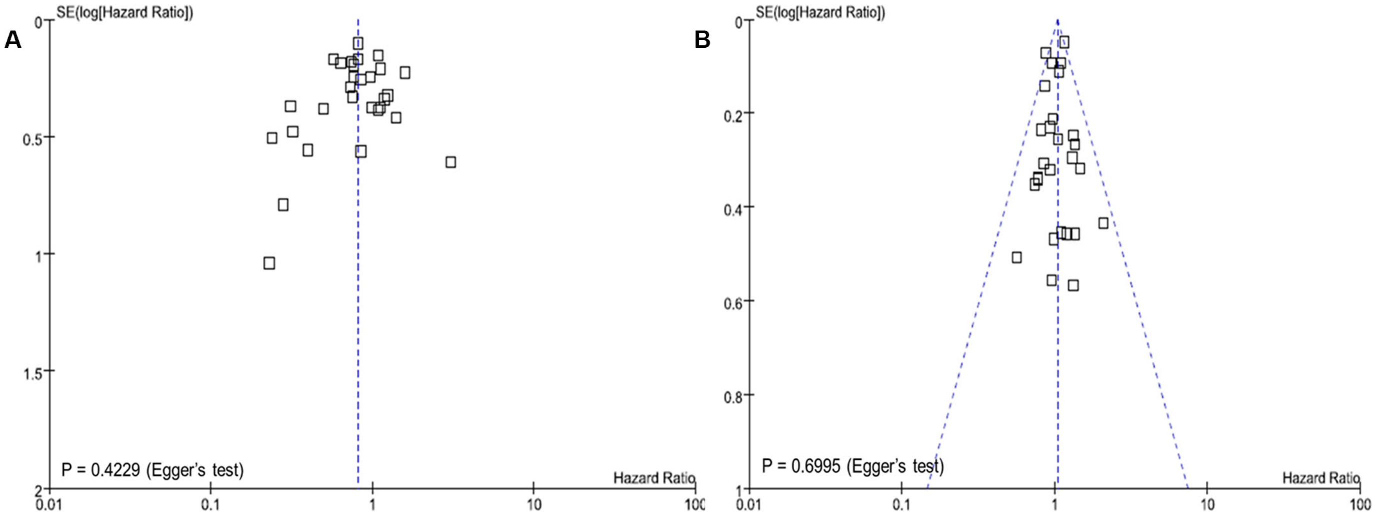

Supplementary Figure S4: Funnel graphs of the assessment of potential publication bias of sex. (**A**) Intravesical recurrence-free survival, (**B**) Cancer-specific survival.

Supplementary Table S1: Pathologic characteristics of the eligible studies. See Supplementary_ Table_S1
