# Supplementary material for: Association between demographic factors and prognosis in urothelial carcinoma of the upper urinary tract: a systematic review and meta-analysis
Source: Oncotarget. 2016 Jul 19;8(5):7464–76. doi: 10.18632/oncotarget.10708 (PMC5352335; doi:10.18632/oncotarget.10708)
Supplement: Supplementary file 2 [file oncotarget-08-7464-s002.docx]

Supplementary Table S1: Pathologic characteristics of the eligible studies

| **Study** | **Pathologic T stage**  **(pTaTis/1/2/3/4)** | **Pathologic N stage**  **(pNx/**−**/+)** | **Tumor grade**  **(G1/G2/G3) or (LG/HG)** | **Lymphovascular invasion** | **Concomitant**  **Carcinoma *in situ*** | **Positive surgical margin** |
| --- | --- | --- | --- | --- | --- | --- |
| Park [11] | 17/20/12/33/4 | 48/27/11 | 6/41/38 | NA | NA | NA |
| Chen [12] | 4/27/23/21/3 | NA | 5/41/32 | NA | NA | NA |
| Ataus [13] | 7/8(T1/2)/9(T3/4) | NA | 8/16 | NA | NA | NA |
| Chung [14] | 80(≤T1)/70(≥T2) | NA | 80/70 | NA | NA | NA |
| Koda [15] | 25/26/17/35/3 | NA | 13/49/44 | NA | NA | NA |
| Akao [16] | 3/24/14/43/6 | 0/79/11 | 4/57/29 | 34 | NA | NA |
| Berger [17] | 28/31/13/24/4 | 82/14/4 | 42/58 | 9 | NA | 7 |
| Li [18] | 101((≤T1)/67/73/19 | 240(NxN0)/0 | 124/136 | NA | NA | NA |
| Soga [19] | 0/013/33/0 | 0/46/0 | 9/29/17 | NA | NA | NA |
| Capitanio [20] | 307/298/237/365/42 | 768/405/76 | 498/751 | 249 | 319 | NA |
| Chung [21] | 9/42/8/17/0 | NA | 2/47/27 | 18 | NA | NA |
| Kamihira [22] | 223/250/164/320/20 | 382/564/57 | 111/522/345 | NA | NA | NA |
| Favaretto (I) [23] | 139(≤T1)/58/77(≥T3) | 93/150/31 | 59/208 | NA | 75 | NA |
| Favaretto (II) [24] | 85/47/56/59/6 | 92/138/23 | 63/190 | NA | 70 | NA |
| Ishikawa [25] | 41/59/44/56/8 | 90/104/14 | 142/66 | 65 | NA | NA |
| Kim [26] | 131(≤T2)/107/0 | 238(NxN0)/0 | 95/143 | 31 | 21 | 10 |
| Kobayashi [27] | 31/58/22/96/7/7/ | NA | 18/117/86 | 73(VI)/60(LI) | NA | NA |
| Pieras [28] | 6/30/21/15/7 | 79(NxN0)/0 | 2/47/30 | NA | 28 | NA |
| Takaoka [29] | 12/13/10/21/3 | 10/46/4 | 31(G1G2)/29(G3) | 20 | NA | NA |
| Cho [30] | 15/29/12/30/1 | 38/39/10 | 16/71 | 21 | NA | 6 |
| Hou [31] | NA | NA | 13/179 | NA | NA | NA |
| Ku [32] | 59(≤T1)/122(≥T2) | 172(NxN0)/9 | 6/38/9 | 48 | 13 | 8 |
| Walton [33] | 172/195/147/215/44 | 590/133/50 | 99/224/450 | 148 | 90 | NA |
| Ariane [34] | 163/144/65/206/31 | 346/209/54 | 50/207/352 | 116 | NA | NA |
| Chen [35] | 5/17/36/16/11 | 66(NxN0)/19 | 31/54 | NA | NA | NA |
| Chromecki [36] | 592/553/473/875(≥T3) | 1675/595/222 | 389/2088 | 597 | NA | NA |
| Godfrey [37] | 78/41/18/71/3 | 152/45/14 | 77/134 | 68 | 132 | 18 |
| Hirano [38] | 23/12/31/69/9 | 111/25/15 | 64/87 | 74 | NA | NA |
| Kobayashi [39] | 57/61/47/112/11 | NA | 155(G1G2)/133(G3) | 88(VI)/89(LI) | NA | NA |
| Kuroda [40] | 6759(≤T2)/54/0 | 114(NxN0)/7 | 49(G1G2)/72(G3) | 35 | 9 | 13 |
| Liang [41] | 181(≤T1)/159(≥T2) | 312(NxN0)/28 | 56/284 | 106 | NA | NA |
| Cho [42] | 15/27/11/25/0 | 32/37/9 | 16/62 | 17 | 17 | NA |
| Ehdaie [43] | 213(≤T1)/75(≥T2) | 260(NxN0)/28 | 65/223 | NA | NA | NA |
| Elalouf [44] | 139(≤T1)/22/76(≥T3) | 147/67/23 | 43/194 | 60 | 50 | NA |
| Fairey [45] | 386(≤T1)/132/188/43 | 617/172/60 | 279/551 | NA | 201 | 82 |
| Fujita [46] | 50/18/11/54/6 | 116(NxN0)/15 | 71/68 | 38 | NA | NA |
| Gunay [47] | NA | NA | 50(G1G2)/46(G3) | NA | NA | NA |
| Hashimoto [48] | 40(≤T2)/44(≥T3) | NA | NA | NA | NA | NA |
| Ito [49] | 16/23/8/25/0 | 32/39/1 | 39/33 | NA | 4 | NA |
| Kim [50] | 2(≤T2)/57/6 | 0/54/11 | 10(G1G2)/55(G2) | 27 | NA | NA |
| Kim [51] | 189(≤T1)/71/152/10 | 381(NxN0)/41 | 208(G1G2)/214(G3) | NA | 33 | NA |
| Kusuda [52] | 218≤T1)/284(≥T2) | 478(NxN0)/24 | 326(G1G2)/176(G3) | 113(VI)/134(LI) | NA | 31 |
| Milojevic [53] | 79(≤T2)/104(≥T3) | 177(NxN0)/6 | 76(G1G2)/107(G3) | 109 | NA | NA |
| Morizane [54] | 47(≤T2)/52(≥T3) | 34/54/11 | 59(G1G2)/38(G3) | 36 | 3 | 4 |
| Rink [55] | 374(≤T1)/186/268/36 | 429/341/94 | 124/740 | 288 | 258 | NA |
| Sakano [56] | NA | NA | NA | NA | NA | NA |
| Shimamoto [57] | 17/18/19/37/14 | 92(NxN0)/13 | 8/37/60 | NA | NA | NA |
| Takahara [58] | 43(≤T1)/13/47(≥T3) | NA | 20/28/55 | 34(VI)/32(LI) | NA | NA |
| Xylinas [59] | 288(≤T1)/53/128/13 | 457(NxN0)/25 | 191/291 | 106 | 28 | NA |
| Zhang [60] | 33/50/28/89/17 | 198(NxN0)/19 | 23/56/138 | 100 | NA | NA |
| Aziz [61] | 106(≤T1)/49/102/8 | 206(NxN0)/59 | 43/60/162 | 52 | NA | NA |
| Bachir [62] | 463(≤T2)/181(≥T3) | 592(NxN0)/52 | 205/439 | NA | NA | 69 |
| Cho [63] | 76(≤T1)/36/35/0 | NA | 47/100 | NA | NA | NA |
| Choo [64] | 130(≤T1)/41/108(≥T3) | 271/42/6 | 228(G1G2)/90(G3) | 60 | 25 | 19 |
| Ehdaie [65] | 132(≤T1)/56/65(≥T3) | 93/137/23 | 59/193 | NA | 70 | NA |
| Fang [66] | 22/131/166/116/3 | 398/34/10 | 15/264/159 | NA | 12 | 0 |
| Fradet [67] | 331(≤T1)/105/182/45 | 571/114/57 | 220(G1)/503(G2G3) | NA | 138 | 58 |
| Fujita [68] | 71/42/40/66/7 | 62/155/9 | 17/105/102 | 45 | NA | NA |
| Gandaglia [69] | 3493(≤T1)/1674/3788/944 | 7561/1545/793 | NA | NA | NA | NA |
| Ichimura [70] | 44/31/18/69/9 | 152(NxN0)/19 | 19/152 | 74 | 83 | NA |
| Ishioka (I) [71] | 303(≤T1)/147/499/31 | 910(NxN0)/104 | 702(G1G2)/287(G3) | 408 | NA | NA |
| Ishioka (II) [72] | 104/133/112/382/23 | NA | 533(G1G2)/221(G3) | 299 | 84 |  |
| Ito [73] | NA | NA | NA | NA | NA | NA |
| Kitamura [74] | 19/19/22/48/0 | 0/108/0 | 3/51/54 | 29 | NA | NA |
| Kondo [75] | 54(≤T1)/25/89/12 | 88/76/16 | 95/85 | 90 | NA | NA |
| Lee [76] | 40/53/73/70/14 | 180/52/18 | 57/193 | 60 | NA | NA |
| Liu (I) [77] | 69(≤T1)/43/98/20 | 183(NxN0)/47 | 57/74/99 | 54 | NA | 19 |
| Liu (II) [78] | 31/81/74/25/1 | 192(NxN0)/20 | 157/55 | 34 | NA | 7 |
| Ou [79] | 9/20/12/19/1 | NA | NA | 9 | NA | NA |
| Park [80] | 81/101/66/135/9 | 213/144/35 | 21/175/196 | 89 | NA | 25 |
| Sasaki [81] | 75(≤T1)/96(≥T2) | 152(NxN0)/19 | 74(G1G2)/97(G3) | 74 | NA | NA |
| Shirotake [82] | 425(≤T2)/414(≥T3) | 787(NxN0)/52 | 332(G1G2)/507(G3) | 314 | NA | NA |
| Sung (I) [83] | 74/89/69/178(≥T3) | 376(NxN0)/34 | 219(G1G2)/182(G3) | 70 | 34 | 21 |
| Sung (II) [84] | 78/85/56/167(≥T3) | 359(NxN0)/27 | 20/194/167 | NA | 30 | NA |
| Tanaka [85] | 159(≤T1)/72/220/23 | 474(NxN0)/0 | 189(G1G2)/285(G3) | 175 | 61 | NA |
| Xylinas (I) [86] | 256(≤T1)/101/140/22 | 260/194/65 | 88/431 | 172 | 175 | NA |
| Xylinas (II) [87] | 1221(≤T1)/519/810/131 | 1662/760/259 | 415/2242 | 579 | 676 | NA |
| Yafi [88] | 0/8/3/240/54 | 177/53/78 | 34/267 | 104 | 75 | 46 |
| Zou [89] | 0/48/48/21/5 | NA | 66/56 | 14 | 10 | 3 |

LG: low-grade, HG: high-grade, NA: not available, VI: venous invasion, LI: lymphatic invasion.
